# Physician communication coaching effects on patient experience

**DOI:** 10.1371/journal.pone.0180294

**Published:** 2017-07-05

**Authors:** Adrianne Seiler, Alexander Knee, Reham Shaaban, Christine Bryson, Jasmine Paadam, Rohini Harvey, Satoko Igarashi, Christopher LaChance, Evan Benjamin, Tara Lagu

**Affiliations:** 1 Department of Medicine, Baystate Medical Center, Springfield, Massachusetts, United States of America; 2 Department of Medicine, Tufts University School of Medicine, Boston, Massachusetts, United States of America; 3 Baycare Health Partners/Pioneer Valley ACO, Springfield, Massachusetts, United States of America; 4 Office of Research, Baystate Medical Center, Springfield, Massachusetts, United States of America; 5 Center for Quality of Care Research, Baystate Medical Center, Springfield, Massachusetts, United States of America; 6 Department of Healthcare Quality, Baystate Medical Center, Springfield, Massachusetts, United States of America; 7 Baystate Health-University of Massachusetts Medical School, Springfield, Massachusetts, United States of America; Yokohama City University, JAPAN

## Abstract

**Background:**

Excellent communication is a necessary component of high-quality health care. We aimed to determine whether a training module could improve patients’ perceptions of physician communication behaviors, as measured by change over time in domains of patient experience scores related to physician communication.

**Study design:**

We designed a comprehensive physician-training module focused on improving specific “etiquette-based” physician communication skills through standardized simulations and physician coaching with structured feedback. We employed a quasi-experimental pre-post design, with an intervention group consisting of internal medicine hospitalists and residents and a control group consisting of surgeons. The outcome was percent “always” scores for questions related to patients’ perceptions of physician communication using the Hospital Consumer Assessment of Healthcare Providers and Systems (HCAHPS) survey and a Non-HCAHPS Physician-Specific Patient Experience Survey (NHPPES) administered to patients cared for by hospitalists.

**Results:**

A total of 128 physicians participated in the simulation. Responses from 5020 patients were analyzed using HCAHPS survey data and 1990 patients using NHPPES survey data. The intercept shift, or the degree of change from pre-intervention percent “always” responses, for the HCAHPS questions of doctors “treating patients with courtesy” “explaining things in a way patients could understand,” and “overall teamwork” showed no significant differences between surgical control and hospitalist intervention patients. Adjusted NHPPES percent excellent survey results increased significantly post-intervention for the questions of specified individual doctors “keeping patient informed” (adjusted intercept shift 9.9% P = 0.019), “overall teamwork” (adjusted intercept shift 11%, P = 0.037), and “using words the patient could understand” (adjusted intercept shift 14.8%, p = 0.001).

**Conclusion:**

A simulation based physician communication coaching method focused on specific “etiquette-based” communication behaviors through a deliberate practice framework was not associated with significantly improved HCAHPS physician communication patient experience scores. Further research could reveal ways that this model affects patients’ perceptions of physician communication relating to specific physicians or behaviors.

## Introduction

Patient experience is an important metric for measuring hospital performance. Since 2012, the Centers for Medicare and Medicaid Services (CMS) have tied patient experience to reimbursement through the Value Based Purchasing (VBP) program. In 2016, 1.75% of a hospital’s Diagnosis Related Group (DRG) base operating payment is at stake and 30% of this payment is linked to a hospital’s patient experience scores.[1] Commercial payers are also linking patient experience outcomes to value-based payments, and many physician groups include patient experience as a metric for physicians’ variable compensation.[2] This increased scrutiny is appropriate, as multiple studies have shown a strong correlation between the quality of physician communication and the quality of clinical care.[3–8] Moreover, many problems with the effective delivery of health care can be attributed to ineffective communication between patient and provider.[9] Practically speaking, good physician communication will be a requirement for hospitals to sustain reimbursement at current levels. Yet there remains a deficit of evidenced-based interventions that lead to improvement of both patient perceptions of these communication skills and overall patient experience.[10–16]

Medical simulation is a validated method of teaching and improving clinical communication abilities. [16–25] Systematic review of clinician communication courses finds an effective approach combines didactic components with simulation with skilled feedback.[24] Programs based upon models of experiential learning and deliberate practice (learning focused on repetitive performance of specified skills with specific feedback)[26] succeed in teaching communications skills and changing clinician behavior. [17,18,25,27,28]

Improvement on clinician communication skills is a prerequisite to improving hospital care. Previous studies show only 10–32% of inpatients can correctly name their physicians, fewer (11%) can explain their physicians’ role in the care that they are receiving, and few can understand the key elements of their hospital plan.[12,29,30] Given these findings, a critical realm to focus improvement on clinician communication relates to basic “etiquette-based medicine” behavior such as knocking on a patient’s door, asking to enter the patient’s room, introducing oneself, sitting down in the patients’ room, and explaining one’s role in care. One cross sectional observational study on such behaviors found that one-third of physicians (30%) performed zero of the six “etiquette-based” behaviors and a majority (56%) did explain their roles to patients, despite the fact that a positive association was found between performance of the behaviors and patient experience scores. [12]

Yet evidence is mixed as to whether clinician communication skills training has an effect on patient satisfaction,[13,14,31] with most interventions showing no effect.[10,13,15,16,32] We hypothesized that using an evidenced-based framework of clinical simulation in a deliberate practice model targeted at teaching simple, discrete physician communication behaviors with structured feedback would improve patients’ perceptions of physician communication and reported experience of care. We implemented such a model in a hospital medicine division at a single academic center and examined its impact on outcomes using two systematically administered patient experience surveys, focusing on domains relevant to physician communication. We compared trends in patient experience scores between medical patients and, where possible, control surgical patients (whose physicians did not undergo the intervention) during the same time period. The control group was included to account for hospital-wide patient experience initiatives occurring concurrent to our intervention time-period.

## Methods

### Setting

Our study was conducted at Baystate Medical Center (BMC), a 716-bed tertiary academic care center in Springfield, MA between January 1, 2011 and December 31^st^, 2013. The hospital has an employed hospitalist group with 50 attending physicians and midlevel hospitalists, 64 internal medicine, and 30 medicine-pediatric residents. The group is separated into two distinct parts, an academic group and a group without resident coverage. Both services care for all adult medicine inpatients and see similar medical patients regardless of insurer, primary care physician, admission diagnosis, or medical comorbidities. Our hospital has surgical services supplied by attending surgical physicians alone as well as by attending supervised surgical house staff, with approximately 75 attending surgeons and 34 surgical residents. Baystate Health’s Institutional Review Board approved this study with a waiver of written consent.

### Study design and intervention

We analyzed two different samples. CMS measures patient experience using a validated tool called the HCAPHs survey.[33] Our primary analysis examined the Hospital Consumer Assessment of Healthcare Providers and Systems (HCAHPS) physician communication domain, which assesses patients’ perceptions of *all of their* hospital physicians’ (e.g., hospitalists, consultants, and trainees) collective communication abilities during the admission. For this analysis, we employed a quasi-experimental design with an intervention group consisting of internists, including hospitalists and residents, and a control group consisting of surgeons. Since HCAHPS data assesses patient perceptions of physician communication not specific to any one clinician, we hypothesized it may not be sensitive enough to assess the effect of a communication intervention focused only on a patient’s hospitalist physician. Therefore, we completed a secondary analysis with a pre-post design utilizing Non-HCAHPS Physician-Specific Patient Experience Survey (NHPPES) data that assessed patient’s perceptions of their specific hospitalist physician’s communication (this survey identifies patient’s individual physician by name, see Survey Questions in S1 Appendix. We were unable to formulate a control group for this secondary sample, as only patients cared for by hospitalists complete the survey.

The intervention was a 45-minute comprehensive physician-training module based on a deliberate practice learning method and proven effective methods of teaching clinician communication skills. The module focused on improving specific “etiquette-based” physician communication skills utilizing simulation and physician coaching with structured feedback. It consisted of a 10-minute didactic lecture highlighting current hospital HCAHPS data results, the rationale behind and importance of performing specific “etiquette-based”[12] basic communication techniques based on the Studor Group’s AIDET^®^ mnemonic,[34] and a structured critique of the performance of these skills utilizing a pre-recorded simulated patient encounter. The structured AIDET-based communication skills specifically targeted were: Acknowledgement (greeting the patient by name, making eye contact), Introductions (introducing oneself by name and clinical role), Duration (giving accurate time expectation for tests and care), Explanations (deliberately explaining what to expect next, patient’s plan of care, and answering questions), and Thank you (appropriately closing the clinical encounter)[34] as well as other non-verbal communication behaviors (body language, facial expressions). Next, individual physicians participated in case—based simulated encounters (approximately 35 minutes in length) focused on the structured communication skills and non-verbal communication behaviors. Prior to participation, each physician received a standardized case scenario (See S2 Appendix) prospectively highlighting the specific skills being assessed, while also varying in complexity according to level of training (Resident versus Attending). The case scenarios broke the simulated patient encounter into three distinct parts: The Welcome, The Care Plan, and The Goodbye. Specific skill based feedback was provided after each part. We undertook a “train-the-trainer” approach where the physician communication champions and the simulated patients underwent a four-hour pre-training of didactic education combined with case-based standardized simulation and feedback on the specific skills of etiquette-based clinician communication and how to provide effective feedback. Personalized physician feedback for each patient encounter was given by both the physician communication champion as well as by the simulated patient after each section of the clinical encounter. A standardized assessment tool (S3 Appendix) was utilized to formulate the focused, structured, personalized, and constructive evaluation and feedback that specifically targeted physician communication etiquette and non-verbal physician behaviors. Of note, other ongoing quality improvement strategies targeted at physicians, including sharing of HCAHPS scores and patient experience email newsletters, continued both before and after the study period.

### Outcome, survey instruments and data collection

The outcome was patient experience scores, collected from adult inpatients admitted before, during, and after the study time period. We collected HCAHPS survey data on both medical and surgical inpatients.

During the study period, Baystate Health used a third party vendor, Professional Research Consultants Inc. (PRC) to administer both the HCAHPS and the NHPPES patient satisfaction telephone surveys to a random sample of discharged adult inpatients. Approximately 50 HCAHPS surveys per quarter, per hospital floor, were conducted and 20 NHPPES surveys per hospitalist per year were conducted among patients not selected for the HCAHPS study. The NHPPES question design included the name of the specific discharging physician who cared for the patient being surveyed and addressed specifics regarding that physician’s care. We limited our analysis to domains reflecting satisfaction with physician communication or those potentially influenced by improved physician communication behaviors.

Survey data were collected for hospital quality and reporting purposes independent of our study. Because the NHPPES survey was only administered to adult medicine inpatients, we collected only HCAHPS data for the control group. The survey responses were scored, depending on question type, with: never, sometimes, usually, always (HCAHPS); or excellent, very good, good, fair, poor (NHPPES). (S1 Appendix)

Additional patient information for respondents was extracted from the hospital’s billing database using medical account numbers and included age, gender, admission year, education level, language, illness severity (the Diagnosis-Related Group severity score, emergency room (ER) admission status, and attending physician type (hospitalist or surgeon). It was not possible to distinguish whether patients were cared for by house staff under an attending physician (medical orders are primarily written by the house staff but patient is rounded on by the entire medical team of house staff and attending) or solely by an attending physician who writes all medical orders and is introduced as the “primary physician”.

### Data analysis

Respondent characteristics were summarized across study groups and time periods. We summarized continuous variables using means and standard deviations and frequencies and percentages to summarize categorical variables. We evaluated differences across groups based on observed differences and a theoretical basis for confounding (association with both the exposure and the outcome). Survey responses were dichotomized into percent excellent (or percent always) and analyzed using a piecewise logistic regression model. All models used a clustered sandwich estimator, clustering on billing physician to relax the assumption of independence of observations.[35] We used piecewise models to estimate a slope and intercept before and after the intervention period with results presented graphically as well as estimates of the average marginal effects (estimated percentages). For the HCAHPS analysis, we used significance testing to evaluate the pre-to-post intercept shift difference-in-difference (interaction term between group and time period). For the NHPPES analysis, we conducted pre-to-post significance testing using intercept shift only. For the primary HCAHPS analysis, initial power calculations suggested that a 0% to 1% pre to post change in the surgery group would give us approximately 80% power to detect a 0.55% to 1.25% difference-in-difference with a two-sided alpha of 0.05. Significance testing was intended to be exploratory in nature; therefore no adjustments for multiple comparisons were made. Multivariable models for both outcomes evaluated age, gender, race, marital status, severity of the patient, preferred language, and admission through the emergency department. The HCAHPS analysis also evaluated education level, overall health and discharge disposition. Models were simplified to contain variables that were significant at the 0.05 level with Wald tests conducted between full and reduced models. Variables that were excluded in this process were entered back into the model, one at a time, and variables that caused approximately a 10% change in the coefficient of interest were retained in the final model. Statistical analysis was conducted using Stata v13.1, StataCorp LP, College Station, TX.

## Results

Of 50 hospitalists, 42 (84%) participated in the physician communication coaching simulation. Of 94 resident physicians, 86 (90%) participated. Hospitalists were 47% female and 53% male, and nearly half (48%) had 0–3 years of attending experience. Only 16% had greater than 10 years experience. The surgical attending clinicians were 16% female and 84% male; we did not have information on years of experience for surgical attendings. The survey participation rate for medicine and surgical patients in the pre-intervention HCAHPS cohort was 31% and 46% and post-intervention 30% and 39%, respectively. The NHPPES physician specific survey participation rate was 22% both pre and post intervention periods.

The HCAHPS patient sample included 5020 patients surveyed (Table 1). The pre-intervention cohort (3720 patients) was surveyed between January 2011 and April 2013 and the post-intervention cohort (1300 patients) was surveyed between June 2013 and January 2014. 33.4% of patients were in the surgical control cohort and 66.7% in the hospitalist cohort. Hospitalist and surgical HCAHPS surveyed patients had similar baseline characteristics. On average, hospitalist patients were slightly older (62.5 years versus 58.5 years), had slightly lower proportion male (46.9% versus 53.2%), lower proportion white (82.8% versus 89.1%), lower proportion with a college degree (16.4% versus 26.3%), and lower proportion English spoken at home (85% versus 93%). Additionally, hospitalist patients were admitted through the ED more often (87.9% versus 27.5%) and had greater disease burden (8.8% versus 4.1% with severity of illness score of 4) but were equally likely to be discharged home or home with services. The HCAHPS hospitalist pre and post-intervention cohorts were similar in all baseline characteristics.

**Table 1:** HCAHPS patient characteristics by provider group over time period.

			Surgery			Hospitalist		
			n = 1674 (33.4%)			n = 3346 (66.7%)		
		Overall	Overall Surgery	Pre	Post	Overall Hospitalist	Pre	Post
		n = 5020	n = 1674 (33.4%)	n = 1263 (75.4%)	n = 411 (24.6%)	n = 3346 (66.7%)	n = 2457 (73.4%)	n = 889 (26.6%)
Age								
	Mean (sd)	61.1 (15.5)	58.5 (14.3)	58.5 (14.1)	58.7 (14.8)	62.5 (15.9)	62.4 (16.0)	62.7 (15.8)
	Median(range)	61 (18–99)	60 (18–95)	59 (18–95)	60 (20–93)	63 (18–99)	63 (18–99)	63 (18–97)
Length of Stay: n(%)								
	Mean (sd)	4.2 (3.7)	4.2 (4.0)	4.3 (4.1)	4.2 (3.6)	4.2 (3.6)	4.2 (3.4)	4.3 (3.9)
	Median(range)	3 (1–46)	3 (1–42)	3 (1–42)	3 (1–21)	3 (1–46)	3 (1–46)	3 (1–34)
	*missing*	*55 (1*.*1)*	*13 (0*.*8)*	*7 (0*.*6)*	*6 (1*.*5)*	*42 (1*.*3)*	*24 (1*.*0)*	*18 (2*.*0)*
Male: n(%)		2459 (49.0)	890 (53.2)	693 (54.9)	197 (47.9)	1569 (46.9)	1156 (47.1)	413 (46.7)
Race: n(%)								
	Black	520 (10.4)	92 (5.5)	71 (5.6)	21 (5.1)	428 (12.8)	302 (12.3)	126 (14.2)
	White	4261 (84.9)	1492 (89.1)	1133 (89.7)	359 (87.4)	2769 (82.8)	2051 (83.5)	718 (80.8)
	Other/Unknown	239 (4.8)	90 (5.4)	59 (4.7)	31 (7.5)	149 (4.5)	104 (4.2)	45 (5.1)
Marital Status: n(%)								
	Married/Partner	2288 (45.6)	945 (56.5)	716 (56.7)	229 (55.7)	1343 (40.1)	978 (39.8)	365 (41.1)
	Single	1630 (32.5)	467 (27.9)	348 (27.6)	119 (29.0)	1163 (34.8)	842 (34.3)	321 (36.1)
	Widowed	558 (11.1)	111 (6.6)	85 (6.7)	26 (6.3)	447 (13.4)	334 (13.6)	113 (12.7)
	Divorced/Separated	537 (10.7)	149 (8.9)	114 (9.0)	35 (8.5)	388 (11.6)	300 (12.2)	88 (9.9)
	Unknown	7 (0.1)	2 (0.1)	0	2 (0.5)	5 (0.2)	3 (0.1)	2 (0.2)
Education: n(%)								
	Less than HS	868 (17.3)	189 (11.3)	135 (10.7)	54 (13.1)	679 (20.3)	479 (19.5)	200 (22.5)
	High School or 2-year Degree	2986 (59.5)	1006 (60.1)	771 (61.1)	235 (57.2)	1980 (59.2)	1477 (60.1)	503 (56.6)
	College or more	988 (19.7)	440 (26.3)	340 (26.9)	100 (24.3)	548 (16.4)	398 (16.2)	150 (16.9)
	*missing*	*178 (3*.*6)*	*39 (2*.*3)*	*17 (1*.*4)*	*22 (5*.*4)*	*139 (4*.*2)*	*103 (4*.*2)*	*36 (4*.*1)*
Overall Health: n(%)								
	Excellent	658 (13.1)	294 (17.6)	210 (16.6)	84 (20.4)	364 (10.8)	260 (10.6)	104 (11.7)
	Very Good	1284 (25.6)	613 (36.6)	469 (37.1)	144 (35.0)	671 (20.1)	474 (19.3)	197 (22.2)
	Good	1493 (29.7)	501 (29.9)	386 (30.6)	115 (28.0)	992 (29.7)	735 (29.9)	257 (28.9)
	Fair	1014 (20.2)	189 (11.3)	147 (11.6)	42 (10.2)	825 (24.7)	621 (25.3)	204 (23.0)
	Poor	462 (9.2)	56 (3.4)	39 (3.1)	17 (4.1)	406 (12.1)	304 (12.4)	102 (11.5)
	No Response	109 (2.2)	21 (1.3)	12 (1.0)	9 (2.2)	88 (2.6)	63 (2.6)	25 (2.8)
English Spoken at Home: n(%)		4407 (87.8)	1556 (93.0)	1180 (93.4)	376 (91.5)	2851 (85.2)	2114 (86.0)	737 (82.9)
	*missing*	*119 (2*.*4)*	*21 (1*.*3)*	*12 (1*.*0)*	*9 (2*.*2)*	*98 (2*.*9)*	*68 (2*.*8)*	*30 (3*.*4)*
Admitted through ED: n(%)		3402 (67.8)	460 (27.5)	336 (26.6)	124 (30.2)	2942 (87.9)	2170 (88.3)	772 (86.8)
Severity: n(%)								
	1	997 (19.9)	559 (33.4)	416 (32.9)	143 (34.8)	438 (13.1)	308 (12.5)	130 (14.6)
	2	1930 (38.5)	719 (43.0)	551 (43.6)	168 (40.9)	1211 (36.2)	901 (36.7)	310 (34.9)
	3	1691 (33.7)	310 (18.5)	222 (17.6)	88 (21.4)	1381 (41.3)	1015 (41.3)	366 (41.2)
	4	364 (7.3)	69 (4.1)	57 (4.5)	12 (2.9)	295 (8.8)	212 (8.6)	83 (9.3)
	*missing*	*38 (0*.*8)*	*17 (1*.*0)*	*17 (1*.*4)*	*0*	*21 (0*.*6)*	*21 (0*.*09)*	*0*
Disposition: n(%)								
	Home Health	1736 (34.6)	715 (42.7)	548 (43.4)	167 (40.6)	1021 (30.5)	740 (30.1)	281 (31.6)
	Home, self	2995 (59.7)	861 (51.4)	641 (50.8)	220 (53.5)	2134 (63.8)	1579 (64.3)	555 (62.4)
	AMA	44 (0.9)	2 (0.1)	1 (0.1)	1 (0.2)	42 (1.3)	34 (1.4)	8 (0.9)
	Hospice	22 (0.4)	4 (0.2)	0	4 (1.0)	18 (0.5)	5 (0.2)	13 (1.5)
	Rehab/SNF	170 (3.4)	83 (5.0)	65 (5.2)	18 (4.4)	87 (2.6)	66 (2.7)	21 (2.4)
	Other Healthcare Institution	51 (1.0)	8 (0.5)	7 (0.6)	1 (0.2)	44 (1.3)	33 (1.3)	11 (1.2)
	*missing*	*1 (0*.*02)*	*1 (0*.*1)*	*1 (0*.*1)*	*0*	*0*	*0*	*0*

Pre and post-intervention patients in the NHPPES survey cohort were similar (Table 2). However, post-intervention patients were slightly younger (58.7 years versus 63 years), had a slightly higher proportion male (53.4% versus 48.4%), were less severely ill (37.8% versus 47.6% with severity of illness score of 3 or 4), and were more likely to have the interview conducted in Spanish (7.7% versus 2.9%).

**Table 2:** NHPPES patient characteristics by time period.

		Overall n = 1990	Pre n = 1638 (82.3%)	Post n = 352 (17.8%)
Age				
	Mean (sd)	62.2 (16.3)	63.0 (16.2)	58.7 (16.7)
	Median(range)	60 (20–96)	64 (18–99)	60 (20–96)
Length of Stay				
	Mean (sd)	4.2 (3.2)	4.2 (3.3)	4.0 (2.9)
	Median(range)	3 (1–29)	3 (1–29)	3 (1–19)
	*Missing*	*33 (1*.*7)*	*28 (1*.*7)*	*5 (1*.*4)*
Male: n(%)		980 (49.3)	792 (48.4)	188 (53.4)
Race: n(%)				
	Black	255 (12.8)	214 (13.1)	41 (11.7)
	White	1636 (82.2)	1343 (82.0)	293 (83.2)
	Other/Unknown	99 (5.0)	81 (5.0)	18 (5.1)
Marital Status: n(%)				
	Married/Partner	870 (43.7)	736 (44.9)	134 (38.1)
	Single	632 (31.8)	494 (30.2)	138 (39.2)
	Widowed	272 (13.7)	235 (14.4)	37 (10.5)
	Divorced/Separated	214 (10.8)	172 (10.5)	42 (11.9)
	Unknown	2 (0.1)	1 (0.1)	1 (0.3)
Severity: n(%)				
	1	319 (16.0)	246 (15.0)	73 (20.7)
	2	751 (37.7)	605 (36.9)	146 (41.5)
	3	775 (38.9)	659 (40.2)	116 (33.0)
	4	138 (6.9)	121 (7.4)	17 (4.8)
	*Missing*	*7 (0*.*4)*	*7 (0*.*4)*	*0*
Admitted through ED: n(%)		1676 (84.2)	1377 (84.1)	299 (84.9)
Interview conducted in Spanish: n(%)		74 (3.7)	47 (2.9)	27 (7.7)

After the intervention, the intercept shift for percent “always” (degree of change from pre-intervention percent excellent responses) for HCAHPS questions of “treating patients with courtesy”, “explaining things in a way patients could understand”, and “overall teamwork” showed no significant differences between surgical control and hospitalist intervention patients either before or after adjustment (adjusted p = 0.899, p = 0.890, p = 0.438, respectively) (Table 3).

**Table 3:** HCAHPS intercept shift by provider group over time period.

		Surgery			Hospitalist			
		n = 1674 (33.4%)			n = 3346 (66.7%)			
Percent Excellent Question		End of Pre	Beginning of Post	Intercept Shift	End of Pre	Beginning of Post	Intercept Shift	*P-value*^a^
		n = 1263	n = 411		n = 2457	n = 889		
		(75.4%)	(24.6%)		(73.4%)	(26.6%)		
Treat you with courtesy & respect								
	Unadjusted	92.5% (89.2–95.7)	93.4% (89.4–97.4)	1.0% (-4.4–6.3)	85.3% (82.2–88.3)	86.4% (82.9–90.0)	1.2% (-3.2–5.5)	*0*.*911*
	Adjusted^b^	92.2% (88.7–95.7)	92.8% (88.1–97.4)	0.6% (-5.4–6.7)	84.5% (81.1–87.8)	86.3% (82.5–90.2)	1.9% (-2.9–6.6)	*0*.*899*
Explain things so you could understand								
	Unadjusted	80.8% (76.2–85.3)	83.6% (78.1–89.1)	2.9% (-4.2–9.9)	72.3% (68.4–76.2)	76.2% (71.0–81.3)	3.9% (-2.0–9.8)	*0*.*983*
	Adjusted^c^	78.1% (72.5–83.7)	80.8% (74.4–87.1)	2.7% (-5.3–10.7)	73.1% (69.2–76.9)	76.9% (71.8–82.0)	3.8% (-1.9–9.5)	*0*.*89*
Overall Teamwork								
	Unadjusted	55.0% (47.9–62.0)	54.4% (46.6–62.3)	-0.5% (-11.2–10.2)	47.9% (44.0–51.9)	43.4% (37.0–49.7)	-4.6% (-12.0–2.8)	*0*.*543*
	Adjusted ^d^	53.2% (46.2–60.3)	54.6% (46.3–62.9)	1.4% (-9.8–12.6)	49.5% (45.5–53.5)	45.6% (39.2–51.9)	-3.9 (-11.4–3.5)	*0*.*438*

^a^ P-value represents X^2^ test evaluating whether the intercept shift in both groups is equal (e.g. difference-in-difference). This p-value is not adjusted for multiple comparisons.

^b^ Adjusted for: age, race, education, health status, language, severity and discharge disposition.

^c^ Adjusted for: race, marital status, education, health status, language, severity, admission through the ED and discharge disposition.

^d^ Adjusted for: marital status, education, health status and language.

**Fig 1:**
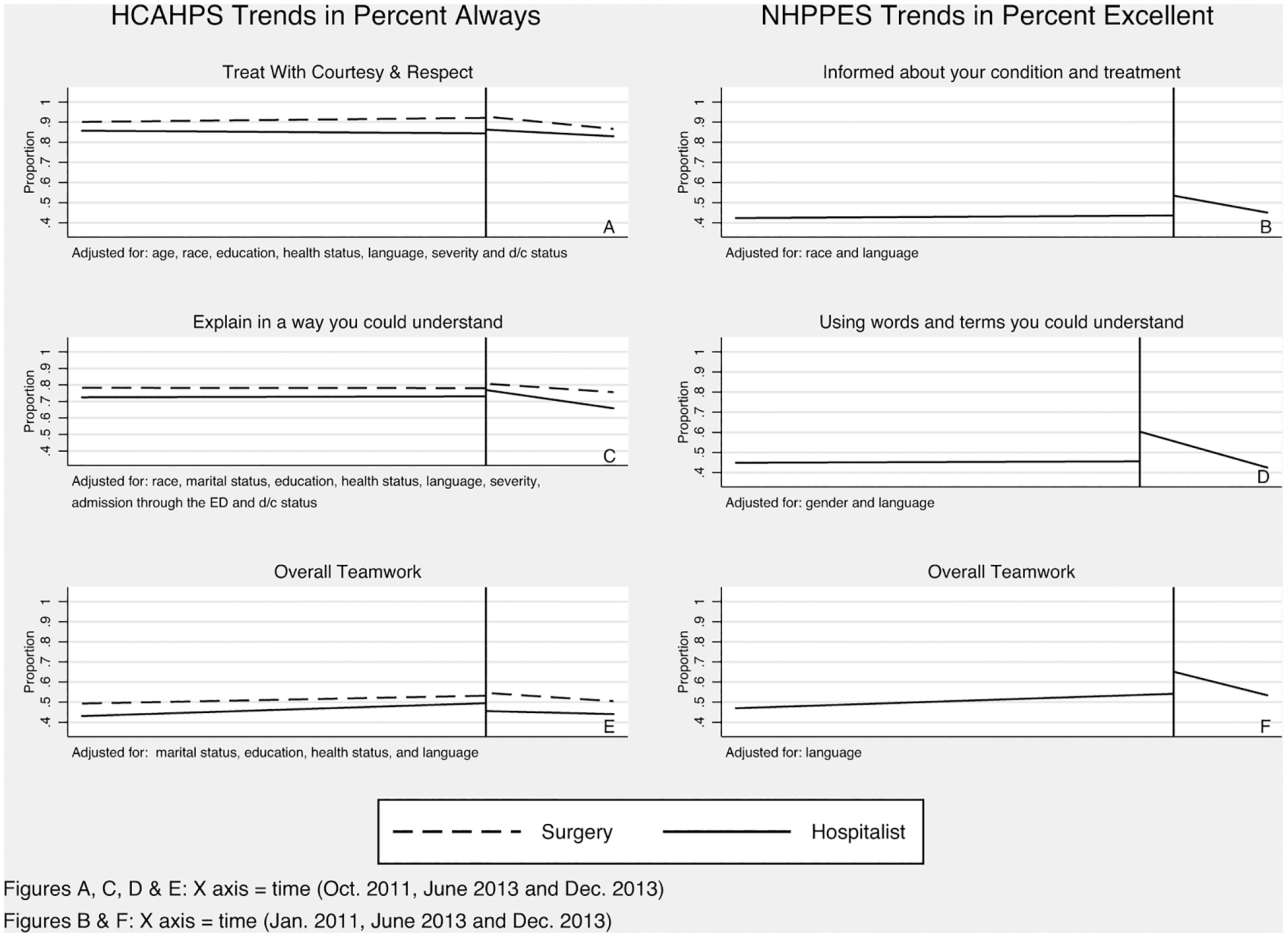
HCAHPS trends in “Percent Always” and NHPPES trends in “Percent Excellent”

NHPPES survey adjusted and unadjusted percent excellent responses increased significantly for the questions of “keeping patient informed” (adjusted intercept shift 9.9% P = 0.019), “overall teamwork” (adjusted intercept shift 11%, P = 0.037), and “using words the patient could understand” (adjusted intercept shift 14.8%, p = 0.001) (Table 4). Post-intervention percent excellent responses increased in the questions addressing “physicians’ explanations of treatments” (adjusted intercept shift 6.9%, p = 0.210) and “treating patients with courtesy” (adjusted intercept shift 3.3%, p = 0.453) but were not statistically significant. Fig 1 shows post-intervention percent excellent responses for all questions analyzed trended towards pre-intervention levels over time (Fig 1).

**Table 4:** Non-HCAHPS physician specific patient experience survey (NHPPES) intercept shift by time period.

Percent Excellent Question		End of Pre	Beginning of Post	Intercept Shift	*P-value*^a^
		n = 1638	n = 352		
		(82.3%)	(17.8%)		
Keeping you informed about your medical condition and treatment					
	Unadjusted	44.7% (39.7–49.6)	54.0% (45.8–62.1)	9.3% (0.4–18.3)	*0*.*042*
	Adjusted^b^	43.6% (38.8–48.4)	53.5% (45.6–61.4)	9.9% (1.6–18.1)	*0*.*019*
Overall teamwork between doctors, nurses and staff					
	Unadjusted	54.9% (50.4–59.3)	65.9% (56.5–75.3)	11.0% (0.7–21.4)	*0*.*045*
	Adjusted^c^	54.2% (49.7–58.6)	65.2% (55.8–74.5)	11.0% (1.0–21.0)	*0*.*037*
Using words and terms you could understand					
	Unadjusted	46.1% (40.1–52.0)	60.5% (54.1–66.9)	14.5% (5.5–23.4)	*0*.*002*
	Adjusted ^d^	45.6% (39.7–51.4)	60.3% (53.8–66.9)	14.8% (5.9–23.7)	*0*.*001*
Instructions or explanations of your treatment or tests					
	Unadjusted	46.9% (40.4–53.4)	53.0% (44.4–61.6)	6.1% (-5.5–17.6)	*0*.*304*
	Adjusted ^b^	46.1% (39.8–52.4)	53.0% (44.8–61.2)	6.9% (-3.8–17.6)	*0*.*21*
Courtesy and friendliness shown to you					
	Unadjusted	57.9% (52.2–63.6)	60.3% (52.6–68.0)	2.4% (-6.6–11.4)	*0*.*6*
	Adjusted ^e^	57.6% (51.8–63.3)	60.8% (53.3–68.4)	3.3% (-5.2–11.8)	*0*.*453*

^a^ P-value represents X^2^ test of the intercept shift. This p-value is not adjusted for multiple comparisons.

^b^ Adjusted for: race and language.

^c^ Adjusted for: language.

^d^ Adjusted for: gender and language.

^e^ Adjusted for: race and marital status.

## Discussion

A hospitalist and IM resident physician communication simulation-based coaching method focused on specific “etiquette-based” communication behaviors through a deliberate practice model with structured feedback was not associated with significantly improved HCAHPS physician communication patient experience scores.

The lack of significant improvement on HCAHPS scores is similar to results from prior physician communication training programs with most showing no effect. [10,13,15,16,32] O’Leary et al. (2013) implemented a three session physician communication skill training program based on AIDET principles for 61 hospitalist physicians in an academic medical center but found no significant improvements in HCAHPS doctor communication domains.[11] However, the O’Leary study also notably showed no significant pre-post improvement in non-HCAHPS (Press Ganey) physician communication questions, while our intervention showed significant improvement in some physician communication questions from a similar non-HCAHPS (NHPPES) patient experience tool. Similarly, a systematic review of physician communication trainings focused on shared decision making showed that only 40% of the studies included showed improve patient satisfaction when providers were trained on communication skills.[13]

Why, then, were there no significant changes in the physician communication domains of HCAHPS scores were seen compared to surgical controls, but our secondary analysis did reveal improvement in similar domains? There are several possible explanations for these findings. First, HCAHPS scores were designed to assess and evaluate patient satisfaction performance in aggregate, at the hospital or system level. [33,36] Although often erroneously used to describe the performance of individual physicians or groups of physicians, our results may illustrate that the HCAHPS survey was not designed to be an evaluation tool of individual provider patient satisfaction performance.[37] In fact, in an analysis of 420 patients admitted to a hospitalist medicine service, the discharging hospitalist accounted for only 34% of all physician encounters. [38] Notably, despite other physicians accounting for the majority of patient care encounters, most performance improvement analyses would attribute HCAHPS outcome data to the discharging hospitalist. Research also shows specialist physicians strongly influence patients overall perceptions of physicians.[39] We also do not know if, for example, hospitalist communication improved but trainees did not similarly benefit. These factors make it difficult to accurately assess the association between HCAHPS physician communication outcomes to individual physician communication behavior or physician communication interventions. A second possible explanation for the difference between HCAHPS and NHPPES results is that fact the HCAHPS analysis examined difference over time compared to control group while the secondary analysis used a weaker, pre-post design (because we lacked an adequate control group). Therefore, while our secondary analysis of NHPPES data suggests that our intervention may have had an effect, we can only conclude that further controlled studies assessing our intervention using a physician specific patient experience survey tool (not HCAHPS) are needed.

Interestingly, despite the limitation of utilizing HCAHPS to access the effectiveness of local level QI activities, a small, single center, non-randomized IM resident education program focused on improving physician related HCAHPS scores did show significant improvements. This intervention linked resident didactic education to individualized patient experience score feedback, monthly recognitions, and tangible incentives.[31] Similarly, our secondary analysis showed improvement in patient reported “excellence” post-intervention, with 10% to 15% increases in percent “excellent” for questions related to keeping patients informed, teamwork, and using understandable patient language. However, the “bump” observed was short-lived, showing physician communication reverts to its prior state after a single interventional period. Unsurprisingly, this suggests personalized physician communication coaching may have some immediate effects that diminish without deliberate reinforcement. This conclusion is supported by Banka et al.’s (2015) study finding significant improvement in HCAHPS scores during their physician communication intervention since they attributed the improvements to ongoing reinforcement activities such as patient satisfaction score feedback, monthly recognition, and incentives for high patient-satisfaction scores.[31] One possible explanation for this finding is that regression towards the mean, a common phenomenon in improvement work requiring behavioral change, occurs if concerted efforts are not made to maintain the educational gain. This leads us to conclude that no single intervention is enough to maintain behavioral improvements over time. Instead, continual reinforcement, perhaps through ongoing simulation training and revisiting the specified skills gained in meetings and through emails, is likely critical to maintain meaningful improvements. [40] However, our intervention represents a typical “real-world” operational quality improvement project where an initial strategy is rolled out but, often due to resource limitations, a similar maintenance strategy is not undertaken. Our study highlights to health systems the critical nature of the “maintenance” phase when determining operational strategies aimed at behavior change.

We believe our intervention, while offering a model of physician communication education and coaching based on evidenced based learning methods, may have also lacked critical change management techniques of real-time data feedback, tangible behavioral incentives, and reinforcement of the specified communication skills. Unsurprisingly, systematic review shows multi-faceted interventions appear to be most effective in quality improvement.[13] However, deciphering which element(s) contribute most to the effect is difficult. Understanding which elements are key in a multi-faceted intervention is critical when adapting successful approaches to health systems with different goals.[13] Therefore, we designed our study to examine the effects of a single intervention. Unfortunately, a more messy “real world” bundled approach (i.e. a “physician communication engagement bundle”) of tactics may be necessary to see significant improvement in HCAHPS scores even if it becomes difficult to decipher which element had the most return on investment. Interestingly, more is not always better. A systematic review of 43 randomized study interventions on physician communication trainings focused on shared decision making found that short-term training (less than 10 hours) is equivalent to longer training.[13] This suggests that some repetition is necessary but that “a lot” more may not be necessary in regards to enhancing physician communication skills.

Educational interventions on physician communication show limited effect on patient experience. [10,13–16,31,32] However, studies do show *the performance* of basic, structured communication behaviors has an association with patient experience outcomes.[12,41] Our results, combined with prior research, still do not fully quantify the utility of structured/scripted communication methodologies as a means to improve patient reported satisfaction with physician communication.[11,41–45] Evidence of scripted communication education improving patient satisfaction remains scarce with most studies being very limited in scope and methodology.[41–45] However, these basic, yet critical skills of “etiquette based” physician-patient communication are rarely done [12] and can lead to serious communication gaps between physicians and their patients. [30]

One small pilot study of 246 emergency room encounters by medical students found the students infrequently (0.4% of encounters) use all targeted communication elements (AIDET framework) but that the use of certain elements (acknowledging a patient by name, explaining that other providers would see the patient) was associated with an increase in patient satisfaction. [41] However, while the utility of scripted patient communication tools is of indeterminate significance on the patient experience, there is ample evidence supporting the educational utility of the learning framework of our intervention. The use of simulation to teach clinical communication skills is well supported in the literature [16–25], as is the utility of teaching communication skills using a model of deliberate practice with structured feedback. [17,18,25,27] We believe our intervention’s communication skills education based on the structured AIDET mnemonic combined with focused feedback mirrors a model of deliberate practice. The structure of the education is valid since a meta-analysis found simulation based medical education with deliberate practice is superior to traditional clinical medical education in achieving specific clinical skill acquisition goal.[46] Likewise, focusing the educational framework on structured “etiquette-based” clinical communication skills is necessary since these behaviors are associated with higher patient experience scores.[12,41] Further research is necessary to fully evaluate the results of the educational framework (utilizing both simulation as well as deliberate practice) on patient reported outcomes. For example, qualitative studies could examine the aspects of communication behaviors that physicians perceived were changed by the training. Controlled studies need to assess our model’s learning framework on a physician specific patient experience. Furthermore, while much research examines the effects of improved “complex” clinician communication skills (such as goals of care discussions) has on the patient experience,[16,20,47] researchers should continue to test the effects on patient experience of the most basic “etiquette-based” elements of physician communication behavior.

Our study has several limitations. We conducted the intervention at a single academic hospital, limiting its generalizability. However, our secondary findings suggest that similar (but repeated) interventions should be studied in more sites, which would allow for control and intervention sites. Most patients who completed surveys were discharged home due to survey administration methods via phone. Similarly, we were unable to assess patient-level differences between survey respondents versus non-respondents. We were unable to determine the number of surveys administered to the patients of each physician. These factors limit generalizability of the study results. However, these limitations are prevalent in most studies utilizing hospital patient satisfaction survey results. The NHPPES survey was administered to only hospitalist patients and we did not have a comparison group for these patients. This poses an avenue ripe for further study. Our study was observational, thus subject to selection bias and confounding. We controlled for identifiable confounders such as illness severity, education, health status, etc. but it is possible that additional unidentified factors could have affected our results. Additionally, we were unable to control for the fact that non-participating hospital medicine physicians did provide some level of hospital care for some of the surveyed patients. This may have minimized our intervention’s significant effect size. We are limited by our use of post-hospitalization surveys, which tend to have a low response rate. Our response rate of 31% for HCAHPS patients is similar to the 2013 HCAHPS national average of 33%.[48] However the NHPPES response rates were appeared to be slightly lower at 22%, which could theoretically introduce bias to our results. Finally, we were limited in that we did not conduct qualitative interviews and did not use other methods to determine how the intervention changed physician behavior. However, we plan to continue to assess this in future studies.

Our research shows the limited utility of a simulation-based, deliberate practice physician communication coaching method with structured feedback to improve physician-related HCAHPS scores. It also highlights the limitations of using HCAHPS to quantify the effects of physician-level patient experience improvement strategies. Secondary analysis results demonstrate potential merit for the educational method focused on “etiquette-based” communication behaviors. Further study of this educational method utilizing controls and a period of behavioral reinforcement tactics (physician communication bundle) needs to be undertaken. Additional research needs to identify validated tools to assess patient perceptions of physician communication as it relates to local physician level quality improvement activities. [37] Without easily executed and validated tools to assess patient experience outcomes at a more granular level, we will never be able to design, implement, achieve and reinforce meaningful change in this arena. Finally, our results suggest that a broader “bundled approach” of multi-faceted physician communication improvement tactics with maintenance strategies may yield more consistent and long-lasting effects.

## Supporting information

S1 Appendix Patient Experience Analysis Questions. (DOCX)

S2 Appendix Observed Structured Clinical Encounter Scenarios. (DOCX)

S3 Appendix Physician Communication Skills Coaching and Assessment Tool. (DOC)
