# Supplementary material for: Physician communication coaching effects on patient experience
Source: PLoS One. 2017 Jul 5;12(7):e0180294. doi: 10.1371/journal.pone.0180294 (PMC5497987; doi:10.1371/journal.pone.0180294)
Supplement: S1 Appendix — (DOCX) [file pone.0180294.s001.docx]

S1 Appendix. Patient Experience Analysis Questions

HCAHPS Patient Satisfaction with Physician Communication Questions

- During this hospital stay, how often did doctors treat you with courtesy and respect?
- During this hospital stay how often did doctors explain things in a way you could understand?
- Would you rate the overall teamwork between the doctor, nurses, and staff as:

NHPS Patient Satisfaction with Physician Communication Questions

- How would you rate [doctor’s name] on keeping you informed about your medical condition and treatment?
- How would you rate [the doctor’s name] on the courtesy and friendliness shown to you?
- How would you rate [the doctor’s name] on using words and terms you could understand?
- How would you rate [the doctor’s name] on the instructions or explanations of your treatments and tests?
- How would you rate the overall teamwork between doctors, nurses, and staff as:
