# Supplementary material for: Physician communication coaching effects on patient experience
Source: PLoS One. 2017 Jul 5;12(7):e0180294. doi: 10.1371/journal.pone.0180294 (PMC5497987; doi:10.1371/journal.pone.0180294)
Supplement: S2 Appendix — (DOCX) [file pone.0180294.s002.docx]

**S2 Appendix. Observed Structured Clinical Encounter Scenarios**

**First Simulation- The Welcome (Acknowledge/Introduce/Manage Up):**

Patient Name: Mr./Mrs. Smith

PCP Name: Dr. Jones

Clinical Scenario:

You (the physician) are meeting the patient (Smith) for the first time in the Emergency Room. You have reviewed the patient’s chart and know they are here for upper GI bleeding. The patient vomited a small amount of blood this morning but they are otherwise clinically stable. Your chart review revealed nothing remarkable. They are being admitted to the hospital (med tele) for further care.

**FOCUS:** You will now **INTRODUCE YOURSELF** to the patient.

You are being evaluated on the following core behaviors:

**ACKNOWLEDGES** the person:

• Knock

• Acknowledge patient by name

**INTRODUCES** and **MANAGES UP** self and colleagues:

• Introduce yourself to patient and others in room (use business card)

• Sit down/be at eye level

• Explain your role as hospitalist/resident

• Explain connection to PCP (and/or hospitalist partners)

• Inform patient you have reviewed chart/familiar w/dx

• BE “ABLE”: Affable + Open/Attentive Body Language + Display Empathy

**Second Section - The Care (Duration/Explanation):**

Patient Name: Mr./Mrs. Smith

PCP Name: Dr. Jones

Clinical Scenario:

You are now picking up right where you left off after completing your introductions with the patient (Smith). As you know, the patient (Smith) vomited a small amount of blood this morning. They are hemodynamically stable and haven’t vomited again since. They do have a history of NSAID use and have never had an upper endoscopy before. They deny any melena or abdominal pain. There labs show a one mg/dL drop in hemoglobin from their baseline but are otherwise unremarkable with a normal coagulation profile. Their exam is benign. They had 100 cc of blood return after an UGI lavage and have had the NGT removed. The patient is still in the ER.

You have now finished taking the history and exam and are ready to **DISCUSS THE PLAN** with the patient.

Your Plan:

- You discussed the patient with Dr. Pleet (GI) and the patient is scheduled to have an endoscopy at 10 am tomorrow.
- They will need to be NPO.
- You have ordered 100cc/hr of saline and for an h/h to be repeated every 8 hours and for IV nexium.
- They are going to a regular medical bed with telemetry.
- You will not be the one rounding on them tomorrow (you are doing another admit shift tomorrow)

**FOCUS:** You will now **DISCUSS THE PLAN** with the patient.

You are being evaluated on the following core behaviors:

**EXPLANATION**

• Clearly explain diagnoses, your care plan, and why in plain terms

• Ask patient if they understand the plan

• Ask patient for permission to proceed with plan

**DURATION:**

• Set expectations for duration/delays of tests

• Set expectations for duration of hospital stay

• Set expectation for when you will explain test results

• Ask patient/family if other concerns

• BE “ABLE”: Affable + Body Language + Empathy

**Third Section: The Goodbye**

Patient Name: Mr./Mrs. Smith

PCP Name: Dr. Jones

Clinical Scenario:

You are now picking up right where you left off having finished explaining your plan to the patient (Smith). The patient expects to be NPO and plans for an upper endoscopy tomorrow am with Dr. Pleet (GI).

You are now ready to END the patient encounter.

You WILL NOT be the physician rounding on the patient tomorrow (you are admitting again tomorrow).

- You need to **REASSURE** the patient and **GIVE CLOSURE** to the patient encounter.

****FOCUS**: You will now **GIVE CLOSURE TO THE PATIENT ENCOUNTER** and move on to rounding on your next admission. **

You are being evaluated on the following core behaviors:

**EXPLANATION:**

• Set expectation for return visit

• Encourage patient to have nurse call if questions

**MANAGE UP:**

• Manage up colleagues

• Manage up nurses/consultants

• Ask pt/family/nurse if any other concerns/needs

• Thank patient for their time, help, patience, etc.

• BE “ABLE”: Affable + Body Language + Empathy
