# Supplementary material for: Physician communication coaching effects on patient experience
Source: PLoS One. 2017 Jul 5;12(7):e0180294. doi: 10.1371/journal.pone.0180294 (PMC5497987; doi:10.1371/journal.pone.0180294)
Supplement: S3 Appendix — (DOC) [file pone.0180294.s003.doc]

| **Key Behaviors:** | **Purpose:** | **Coach Assessment: Place an X in Boxes below, with justifying comments.**  **1 = Did Not Demonstrate**  **2 = Demonstrates Competence**  **3 = Exceptional use of Communication Behavior**  ****Please Assess Body Language and Non-Verbal Communication Behavior**** | | | |
| --- | --- | --- | --- | --- | --- |
|  |  |  |  |  |  |
|  |  |  |  |  |  |
|  |  | **1** | **2** | **3** | **Notes** |
| **Phase #1: The Introduction** | **GOAL:** Establish rapport and trust by showing courtesy, attention, and offering explanations |  |  |  |  |
| **ACKNOWLEDGE** the person:   - Review the chart before entering; inform pt. - Knock and acknowledge patient by name - Sit down, make eye contact | - Patient sees you are prepared and interested - Shows courtesy and verifies identity - Patient sees you want to listen | ****  ****  **** | ****  ****  **** | ****  ****  **** |  |
| **INTRODUCE and MANAGE UP** self and colleagues:   - Introduce yourself (Use Business Card) - Explain your role as hospitalist - Explain your connection to PCP | - Patient knows who you are - Patient knows why you are there - Patient assured that PCP will be “in the loop” | ****  ****  **** | ****  ****  **** | ****  ****  **** |  |
| **Phase #2: The Care Plan** | **GOAL:** Solidify trust and understanding of care plan and what to expect to encourage shared decision making |  |  |  |  |
| **EXPLANATION:**   - Clearly explain your care plan and why - Assess patient understanding of care plan - Assess for agreement (Shared decision making) | - Patient is informed and knows what to expect - Patient can ask questions or raise concerns - Patient preferences play a role in care | ****  ****  **** | ****  ****  **** | ****  ****  **** |  |
| **DURATION:**   - Set expectations for duration or delays - Set expectation for duration of hospitalization - Set expectation for when you will return | - Patient knows what to expect | ****  ****  **** | ****  ****  **** | ****  ****  **** |  |
| **Phase #3: The Goodbye** | **GOAL:** Maintain trust with appropriate closure of interaction with clear explanations and expectations. |  |  |  |  |
| **EXPLANATION:**   - Set expectation for return visit - Describe team nature of inpatient care - Describe how nurse can always contact you   **MANAGE UP:**   - Manage up the nurses, case managers, specialists, and healthcare team - Give assurance you will coordinate care - Manage up your hospitalist teammates | - Patient knows when you will return - Patient knows you are available if needed - Reinforces team culture; builds confidence in team - Reduces patient anxiety - Patient knows what to expect, when, and who is part of their “team” | ****  ****  ****  ****  ****  **** | ****  ****  ****  ****  ****  **** | ****  ****  ****  ****  ****  **** |  |
